# Boosting brackish water treatment via integration of mesoporous γ-Al_2_O_3_NPs with thin-film nanofiltration membranes

**DOI:** 10.1038/s41598-022-23914-2

**Published:** 2022-11-16

**Authors:** Gamal K. Hassan, Mona Al-Shemy, Abeer M. Adel, Aly Al-Sayed

**Affiliations:** 1 Water Pollution Research Department, National Research Centre, 33 El Buhouth St. (Former El-Tahrir St.), P.O. Box 12622, Dokki, Giza Egypt; 2 Cellulose and Paper Department, National Research Center, 33 El-Bohouth St. (Former El-Tahrir St.), P.O. 12622, Dokki, Giza Egypt

**Keywords:** Chemistry, Polymer chemistry, Nanocomposites

## Abstract

In this study, a simple method based on non-ionic surfactant polysorbates-80 was used to create mesoporous γ-Al_2_O_3_NPs. The properties of the prepared mesoporous alumina nanoparticles (Al_2_O_3_NPs) were verified using ATR-FTIR, XRD, SEM, TEM, DLS, and BET surface area analysis. Then, thin-film nanocomposite (TFN) nanofiltration membranes were fabricated by interfacial polymerization of embedded polyamide layers with varied contents (0.01 to 0.15 wt.%) of mesoporous γ-Al_2_O_3_NPs. The surface roughness, porosity, pore size, and contact angle parameters of all the prepared membranes were also determined. The performance of the fabricated membranes was investigated under various mesoporous γ-Al_2_O_3_NPs loads, time, and pressure conditions. Mesoporous γ-Al_2_O_3_NPs revealed an important role in raising both the membrane hydrophilicity and the surface negativity. The addition of 0.03 wt.% mesoporous γ-Al_2_O_3_NPs to the TFN membrane increased water flux threefold compared to the TF control (TFC) membrane, with maximum water flux reaching 96.5, 98, 60, and 52 L/(m^2^.h) for MgSO_4_, MgCl_2_, Na_2_SO_4_, and NaCl influent solutions, respectively, with the highest salt rejection of 96.5%, 92.2%, 98.4%. The TFN-Al_2_O_3_ membrane was also able to soften water and remove polyvalent cations such as Mg^2+^ with a highly permeable flux. The TFN-Al_2_O_3_ membrane successfully removed the hardness of the applied water samples below the WHO limit compared to using merely the TFC membrane. Furthermore, the TFN-Al_2_O_3_ nanofiltration membrane unit proved to be a promising candidate for the desalination of real brine like that collected from the Safaga area, Egypt.

## Introduction

The lack of fresh water is a major global problem for many countries, especially developing and poor countries. That is why the research has always been for advanced desalination technologies that have a small footprint, low energy consumption, low cost, and high purification efficiency, in addition to being environmentally friendly. Among all the technologies that have recently emerged, membrane frameworks are the best and most widespread in the field of study to achieve all the required qualities mentioned above^1–3^. Nanofiltration (NF) membranes are the most widely deployed separation technology in the world due to their ability to get rid of various minerals present in different types of water to produce high-purity water that can be reused again. The nanofiltration units are characterized by the dimensions and performance of pores located between reverse osmosis (RO) and ultrafiltration (UF) units, which makes them mainly qualified for the removal of binary salts and organic molecules with an atomic mass unit ranging from 200 to 1000 Da. It also differs from RO units in that it requires low operating pressure^4,5^.

Additionally, brackish water, which makes up more than half of the world's groundwater, has been treated using these membranes^6–8^. For academics and decision-makers, the value of utilizing brackish water as an unconventional water resource is increased by this global percentage of brackish water. However, treated brackish water only made up 22% of the water treated using innovative desalination technologies^9^. This motivated scientists to apply innovative alterations to the NF membranes that are employed in the desalination of brackish water.

Recently, cellulose acetate and polyamide (PA) polymers are the most widely used in the fabrication of NF membranes. However, PA membranes are more popular than cellulose acetate membranes because they avoid many of the defects caused by the utilization of cellulose acetates, such as a narrow pH range and lack of resistance to microbial erosion^10^. Although PA-NF membranes can be manufactured by both interphase polymerization and phase blending techniques, the interfacial technique is the best in providing superior selectivity and advanced flow permeability^11,12^. The thin functional layer of PA is fixed on a thick and permeable substrate of polysulfone. The active segregation ability of the PA and the polysulfone base improves the efficacy of the thin-film (TF) membrane^13^. To further improve the performance of the TF membrane, the addition of metal–organic frameworks and inorganic nanomaterials (such as carbon nanotubes, silicon dioxide, zeolite, titanium dioxide, graphene oxide, and alumina) to the PA matrix layer can result in roughness reduction and improved water flux and hydrophilicity^14^.

Asadi et al.^15^ have developed a new nanofiltration membrane based on polyethersulfone amended by mesoporous carbons to remove various dyes and salts. They reported a higher rejection rate from the bare nanofiltration membrane (up to 64% for CaCl_2_ and 95% for Na_2_SO_4_), increased flux permeability to 20.11 kg/(m^2^.h), and higher rejection values for various dyes (up to 90%). Lai and co-workers also fabricated chitosan-based mesoporous silica thin films with superior nano-filtration membranes for the purification of dye wastewater^16^. They stated that the rejection of Congo Red dye was more than 98% with the superior performance of the used membrane. Recently, alumina nanoparticles (Al_2_O_3_NPs) has been used in many applications such as pharmaceuticals, abrasives, medical implants, as well as electrical insulators due to their unique physical and chemical properties of high hardness, stability, insulation, and transparency^17,18^. Al_2_O_3_NPs can be used as well as additives or fillers in polymers to improve tensile strength, fracture toughness, and corrosion strength, as for epoxy polymers^19,20^. The two phases of alumina are alpha (α) and gamma (γ). The main distinction between these two phases is that α phase, which is solid alumina, has higher heat conductivity than the γ phase, which is porous alumina. Because α phase is denser than the γ phase, the conductance difference is rational. Additionally, while γ-alumina is a porous alumina and has a large surface area with acidic properties, α-alumina has a very low surface area and is virtually completely impermeable. As a result, the γ phase is frequently utilized as catalyst support for the adsorption of different metals or metal oxides to offer catalytic properties, while α phase is utilized as a ceramic material^21,22^. Hence, based on the differences in surface area and activity between the two phases, the γ phase would be significantly more suitable for the development of TF nanocomposites (TFN) membranes. Several previous studies investigated the incorporation of γ-Al_2_O_3_NPs into nanofiltration membranes via interfacial polymerization or phase inversion to improve their desalination performance^23–27^. However, nanofiltration with thin films amended with mesoporous γ-Al_2_O_3_NPs is still lacking.

Biosynthesis, pyrolysis, laser ablation, gel distribution, ball milling, hydrothermal distribution, and gibbsite dissociation of Al(OH)_3_ are all methods for producing Al_2_O_3_NPs^28–30^. Recently, surfactant molds for NP synthesis have gained much interest as it has been possible to obtain NPs from micelle solutions of ionic surfactants using the metal ion to be shaped as NPs as the opposite ion. The ionic surfactants act as stabilizers to sustain the dispersion of NPs, while the controlling mechanisms are hydrogen bonds and electrostatic interactions^31^. Although it is possible to obtain a more stable dispersion when the system contains an opposite charge of NPs and surfactants with minimal zeta potential (20 mV), it has been reported that nanosuspensions with zeta potential below (20 mV) could form a physically stable dispersion with non-ionic surfactants, resulting from their steric impact. Non-ionic surfactants can be dissolved without being ionized by weak hydrophilic groups such as hydroxyl groups and ether-type bonds. The non-ionic surfactants are the most widely used stabilizers in the industry and, by their chemical and physical properties, have an impressive effect on NP systems. Nevertheless, regardless of their broad applications, new properties and recruitment patterns of polysorbates are still being uncovered and reported at present^32^.

In this study, polysorbate-80 is used as a template to prepare mesoporous γ-Al_2_O_3_NPs with distinctive properties.

Following this, the fabrication of the novel TFN membrane, which has till now not been studied, is carried out by introducing the synthesized mesoporous γ-Al_2_O_3_NPs into the active PA layer via interphase polymerization. This technology allows the development of an efficient and feasible desalination method with significantly enhanced nanofiltration performance to remove divalent ions. Moreover, the properties of different TFN-Al_2_O_3_ membranes containing different weight ratios of mesoporous γ-Al_2_O_3_NPs are investigated (the synthesis is included in the organic trimesoyl chloride solution during the interphase polymerization process). The study of water flux and different salts' rejection across the newly manufactured membranes is then compared to a TF control (TFC) membrane. The membrane with the optimum efficacy is also specified by the performance testing. Finally, the improved nanofiltration membranes are applied in the desalination processes of brackish groundwater collected from the Safaga area, Red Sea coast, Egypt.

## Experimental

### Materials

Aluminium nitrate and ammonium hydroxide solution (NH_4_OH) were purchased from Merck Chemicals Co., Darmstadt, Germany. Polysulfone was purchased from Solvay advanced polymers, USA. M-phenylenediamine (99.4%), n-hexane (high purity, 97%), Polysorbates-80 (polyoxyethylene sorbitan monooleate, Tween-80), Trimesoyl chloride (98.5%), Sodium laurylsulfate (SLS, CH_3_(CH_2_)_11_SO_4_Na), Na_2_SO_4_, MgSO_4_, MgCl_2_, and NaCl were all brought from Sigma-Aldrich. All solvents used in this work were of high purity acquired from Sigma-Aldrich. Other chemicals in the present study were of analytical grade utilized as it is without any further purification.

### Fabrication of mesoporous alumina nanoparticles (Al_2_O_3_NPs) using polysorbates

**Figure 1:**
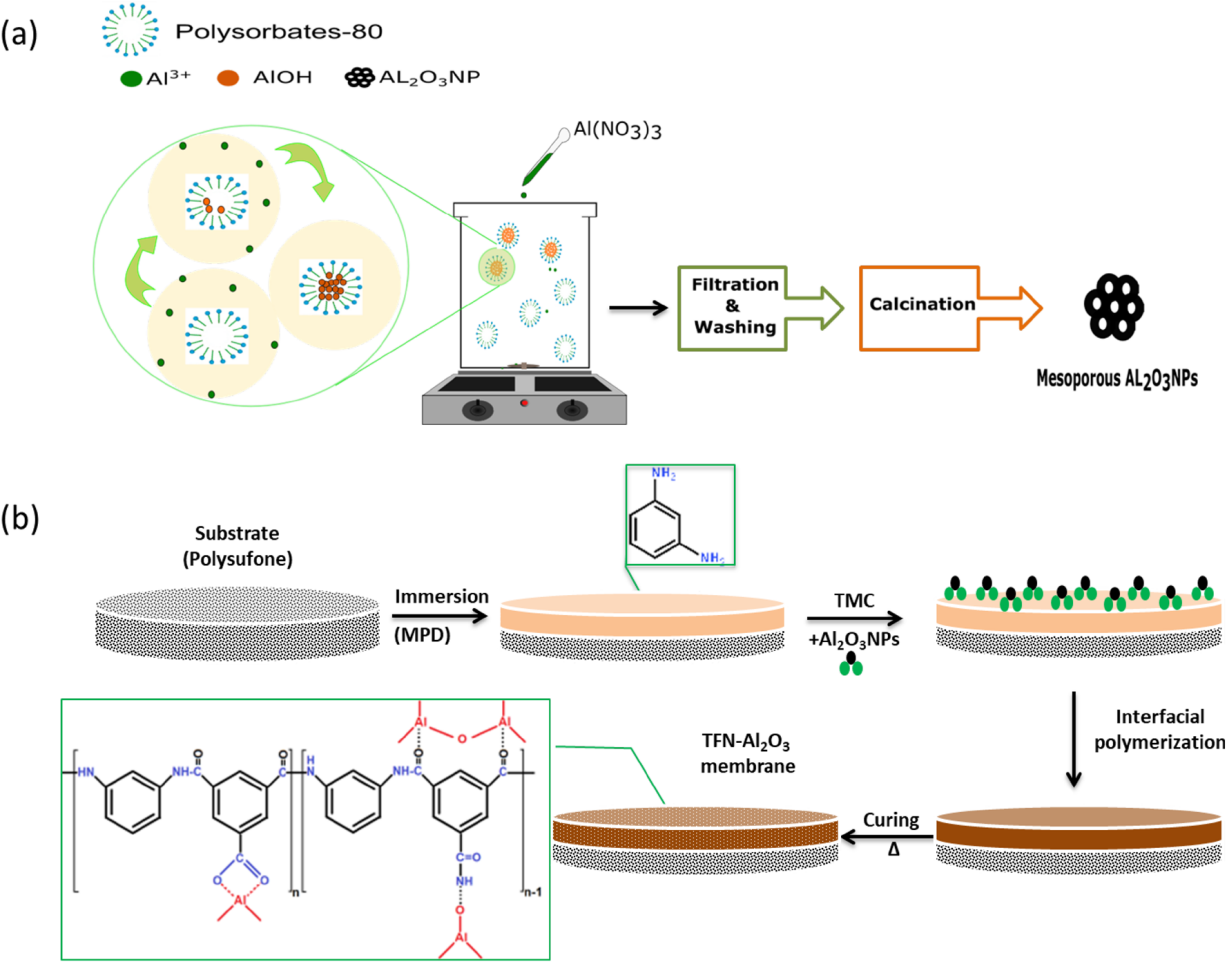
Fabrication steps of (**a**) mesoporous γ-Al_2_O_3_NPs and (**b**) TFN-Al_2_O_3_ membranes.

Mesoporous Al_2_O_3_NPs were prepared by drop wise adding of 0.3 M Al(NO_3_)_3_, precursor, to 0.06 M Polysorbates-80 at pH 9.1 (using ammonium hydroxide solution) and 25 °C for 2 h until white precipitate was formed. The precipitate was centrifuged and washed with deionized water (DI) several times, and then the collected precipitate was oven dried at 80 °C for 24 h. The final product was calcined at 800 °C for 5 h after which, it was packed for further characterization and application in TFN membrane. Figure 1a illustrated the fabrication steps of mesoporous γ-Al_2_O_3_NPs.

### Fabrication of TFN membranes

To prepare TFN membranes, a pristine thin-film membrane was made through interfacial polymerization on a polysulfone support sheet. The support sheets were made by coating a non-woven fabric support with an 18% polysulfone polymer solution in DMAc solvent through a molding blade to a wet thickness of 100 μm. After washing the polysulfone sheets various times with DI, they were kept for one day before use. The polysulfone uphold sheet, positioned on a glazier lid, was dipped for two minutes in 50 ml aqueous solution containing 2% M-phenylenediamine (MPD) and 0.15% sodium laurylsulfate. After the polysulfone layer was saturated with M-phenylenediamine, the sheet was robbed by a rubber drum to eradicate leftover M-phenylenediamine solution. Next, the saturated polysulfone support sheet was soaked for one minute in 50 ml of 0.15% of trimesoyl chloride (TMC)/n-hexane solution. To prepare TFN membranes, varied fractions of the fabricated mesoporous γ-Al_2_O_3_NPs (0.01, 0.03, 0.06, and 0.15 wt.%, based on the weight of trimesoyl chloride monomer) were added to the trimesoyl chloride organic solution. For uniform distribution, the mixture with mesoporous γ-Al_2_O_3_NPs was homogenized using sonication probes. The ensuing membranes were fixed for 10 min at 80 °C (see Fig. 1b). Then, TFC and TFN membranes were reserved in bi-DI water for further characterization and brackish water treatment applications.

### Characterizations

The particle size distribution and high-resolution transmission electron micrograph of prepared mesoporous γ-Al_2_O_3_NPs were recorded on a dynamic light scattering DLS (Malvern Zetasizernano series) and high-resolution JEOL JEM-2100 microscopy, respectively. The nitrogen adsorption–desorption isotherm was measured on the fabricated mesoporous γ-Al_2_O_3_NPs using the Quantachrome TouchWin Instrument to determine their porous structure and average particle size. The Brunauer–Emmett–Teller (BET), density functional theory (DFT), and Langmuir methods were applied for calculations of average surface area. Fourier Transform Infrared spectroscopy (Shimadzu FT-IR-8400 S, Japan), in the range of 4000–400 cm^−1^ with a resolution of 4 cm^−1^ and an average of 32 scans, was used to investigate the chemical functional groups of fabricated mesoporous γ-Al_2_O_3_NPs and TF membranes. The X-ray diffraction profiles of the prepared membrane samples were recorded on a Panalytical Empyrean X-ray diffractometer. Environmental scanning electron microscopy (Quanta FEG-250 microscope) was carried out to analyze surface morphology and EDX analysis of fabricated mesoporous γ-Al_2_O_3_NPs and TF membranes. The surface zeta potentials of the membrane samples were measured with a Nicomp ZLS380, USA, using an aqueous solution of 0.001 M KCl at pH 7.0 and 25.0 °C. For the different fabricated membranes, the roughness of surfaces was explored through the Non-Contact mode of atomic force microscopy (Shimadzu Wet-SPM9600, Japan). In addition, the average contact angle of prepared membranes was measured by a video contact angle system (Kr ÜssDSA25B, Germany). The water drops on five different places of each membrane surface were utilized to investigate the average surface hydrophilicity. The mechanical strength properties of the fabricated membranes were evaluated via the LLOYD LR 10 K universal testing machine. The test strips were cut into rectangular shapes of 70 mm in length by 15 mm in width. The gauge length was fixed at 20 mm. The results are average values of five repeated tests on each membrane. Finally, the conductivity meter (Hanna devices, Ann Arbor, Michigan, USA) was used to estimate the electrical conductivity of the salt solutions.

### Evaluations of TFN-Al_2_O_3_ membrane performance

The separation performance of the pristine TFC and TFN-Al_2_O_3_ membranes in terms of salt rejections and water permeability was held according to^33^. To stimulate the fixed flux of the nanofiltration membranes, the cross-stream appliance was supplied with DI water before operating at 10 bar for 24 h. Subsequently, the water permeability was examined at 10 bar for an hour (Eq. 1).

1 $$J = \frac{V}{A* t*p}\;\left( {{\text{L}}/\left( {{\text{m}}^{{2}}.\;{\text{h}}} \right)} \right)$$

Here, the membrane active area (*A*, m^2^), the permeate water volume (*V*, L), the applied pressure (*P*, bar), and the running time (*t*, h)^34^.

A 2 g/L single salt solution of (NaCl, Na_2_SO_4_, MgSO_4_, and MgCl_2_) was used for the rejection experiments at an applied pressure of 10 bar. At 25 °C, an across-flow rate of 0.22 m/s was used to complete the rejection test. From Eq. (2), the rejection percentage (R %) can be estimated as follows:

2 $$R = \left( {1 - \frac{{C_{P} }}{{C_{f} }}} \right)100\%$$

Here, the concentrations of permeate (*C*_*p*_) and feed solutions (*C*_*f*_)^35^.

### Performance of TFC and TFN-Al_2_O_3_ membranes against an actual sample of brackish groundwater

The actual sample of brackish groundwater was brought from the Safaga area, Suez Governorate, Egypt. The pH value was measured by the JENWAY 3510 device, and the total dissolved solid (TDS) value was measured by the JENWAY 4510 device. Calcium, magnesium, sodium, and potassium concentrations in brackish water were determined after filtration through a 0.22 µm membrane filter (Thermo Fisher, USA) utilizing ion chromatography (ICS 5000 + , Dionex Corporation, USA). The bicarbonate, carbonate, chlorine, and sulfate ions were measured based on the methods described by APHA^36^. In addition, retention coefficients (*R*_*salinity%*_) can be calculated using Eq. (3):

3 $$R_{Salinity\% } = \left( {\frac{{\left( {A_{influent} - A_{effluent} } \right)}}{{A_{influent} }}} \right) \times 100$$

Here, the records of electrical conductivity for influent (*A*_*influent*_*)* and effluent *(A*_*effluent*_*)* of the water streams^37^.

Eventually, the hardness rejection degree (*R*_*Hardness%*_) was computed according to Eq. (4):

4 $$R_{Hardness \% } = \left( {\frac{{\left( {H_{influent} - H_{effluent} } \right)}}{{H_{influent} }}} \right) \times 100$$

Here, the hardness amounts of influent water stream (*H*_*influent*_) and effluent water stream (*H*_*effluent*_)^38^.

### Statistical analysis

At least three durations were implemented for all membrane performance experiments. Statistics were accomplished by (ANOVA) and SPSS 13.0 (p < 0.05). Investigations were performed after two (GLM) to assess the distinctions in the removal percentage of chloride ions, sulfate ions, hardness, and salinity in an actual brackish water sample between five different membranes.

## Results and discussion

### Characterization of fabricated mesoporous γ-Al_2_O_3_NPs

**Figure 2:**
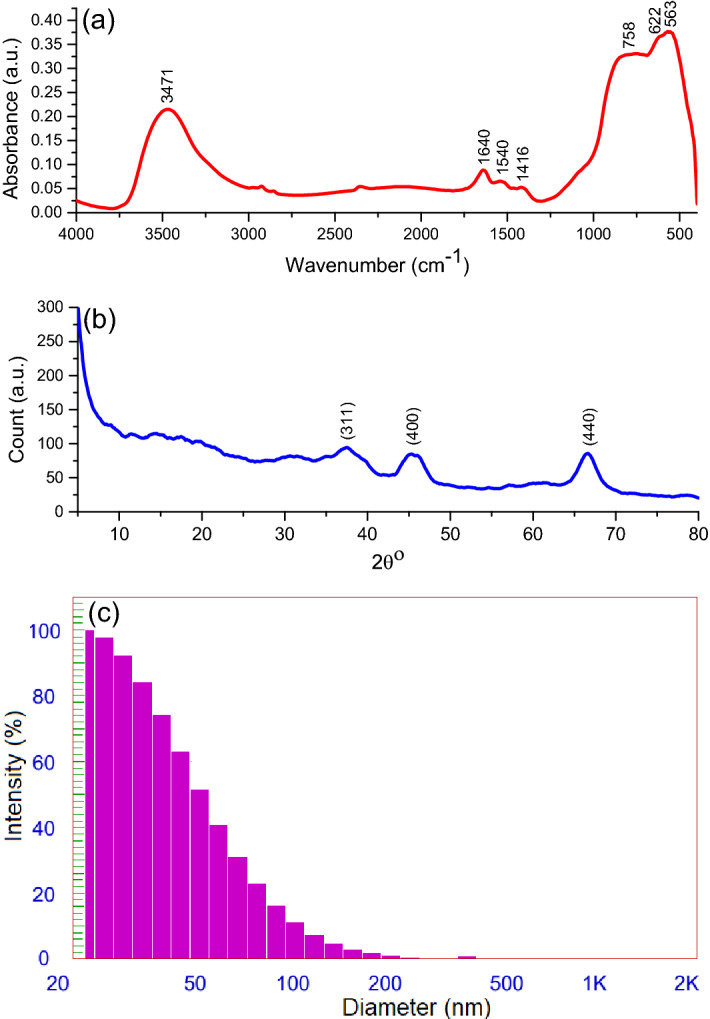
(**a**) FTIR spectrum, (**b**) XRD spectrum, and (**c**) particle size distribution of fabricated mesoporous γ-Al_2_O_3_NPs.

The FTIR, XRD, and DLS analyses of the fabricated Al_2_O_3_NPs are shown in Fig. 2. The prominent absorption peak at 3471 cm^−1^ in the FTIR spectrum (Fig. 2a) is characteristic of the OH stretching vibrations from the Al–OH moieties of mesoporous γ-Al_2_O_3_NPs. Furthermore, the peaks below 1000 cm^−1^, caused by asymmetric and symmetric stretching and binding vibrations of Al–O–Al bonds, confirm the structure of Al_2_O_3_NPs' γ-phase^39,40^. In addition, both the octahedral and tetrahedral coordination of the γ-phase of Al_2_O_3_NPs can be inferred from the shoulder that appeared at 758 cm^−1^, the line at 950 cm^−1^ for Al^IV^, and the peaks at 563, 622, and 758 cm^−1^ assigned to Al^VI^ ^41^. However, the observation of three absorption peaks at 1640, 1540, and 1416 cm^−1^ can be an indication of the potential covalently bonded carboxylate groups (comprise bridging carboxylates) covering the Al_2_O_3_NPs surface^42^. Accordingly, after calcination at a temperature of 800 °C, carboxylate groups’ remains, and this consequently forms organic substituted Al_2_O_3_NPs. These results are consistent with what was obtained in an earlier work where alumina nanoparticles were synthesized eco-friendly via colophony extract. The prevalence of these carboxylate groups at this temperature may be credited to the complexity of the polysorbate structure^43^.

XRD analysis is another tool utilized to find the phase structure of the fabricated mesoporous γ-Al_2_O_3_NPs. Figure 2b displays the XRD spectrum with three diffraction peaks at the interplanar spacing of 2.42, 1.98, and 1.40 Å characteristic of the Miller index planes (311), (400), and (440), respectively, for the γ-phase of Al_2_O_3_ according to JCPDS card: 100425^44,45^. This XRD result is consistent with the outcomes of the FTIR analysis, where the γ-phase was identified. The average crystalline size of the γ-phase determined at an interplanar spacing of 1.40 Å using the Debye Scherrer equation was 15.13 nm^46^. Also, the XRD spectrum presents an amorphous segment which denotes the extreme dehydration of the surface O–H groups. This process relies on indistinct spinal configurations, which leads to the creation of a γ-phase is established by the site of the Al^3+^ ions as either tetrahedral or octahedral positions inside the spinel configuration^47,48^. Thus, the XRD configuration with significant broad and dispersed diffraction peaks indicates the compositional variations and occurrence of slight crystalline particles.

Moreover, the particle size distribution, as was found by DLS analysis, shows an average particle size of 28.9 nm, which confirms that the prepared NPs fell in the required range before being used in this study (Fig. 2c).

**Figure 3:**
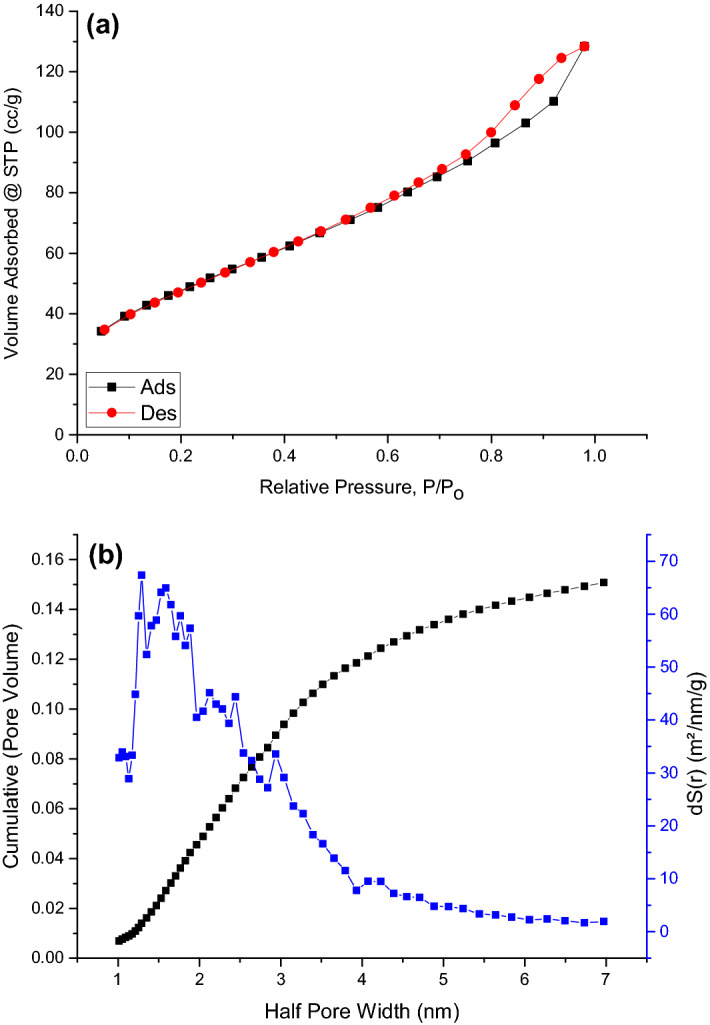
Nitrogen physisorption isotherm and DFT method of fabricated mesoporous γ-Al_2_O_3_NPs.

Figure 3 shows a summary of the results for surface area, pore volume, and pore diameter as calculated from the nitrogen physisorption isotherm. The surface area as per the BET (S_BET_), Langmuir (S_Langmuir_), and density functional theory (DFT) (S_DFT_) methods are 172 m^2^/g, 255.56 m^2^/g, and 143.458 m^2^/g, respectively, while the average particle diameter and pore diameter are 15.87 nm and 4.63 nm, respectively. This high surface area indicates a relatively high porosity. From the classification adopted by the International Union of Pure and Applied Chemistry (IUPAC), materials can have either micropores if the pores' mean diameter is less than 2 nm, mesopores if the mean pore diameter is in the range of 2 nm to 50 nm, or macropores if the mean pore diameter is > 50 nm, and thus the fabricated γ-Al_2_O_3_NPs, with an average pore diameter of 4.63 nm, can be classified as mesoporous nanoparticles^49^. This can also be inferred from the IV-type of nitrogen physisorption isotherm and the decrease in nitrogen uptake under a relative pressure of 0.4^50,51^.

**Figure 4:**
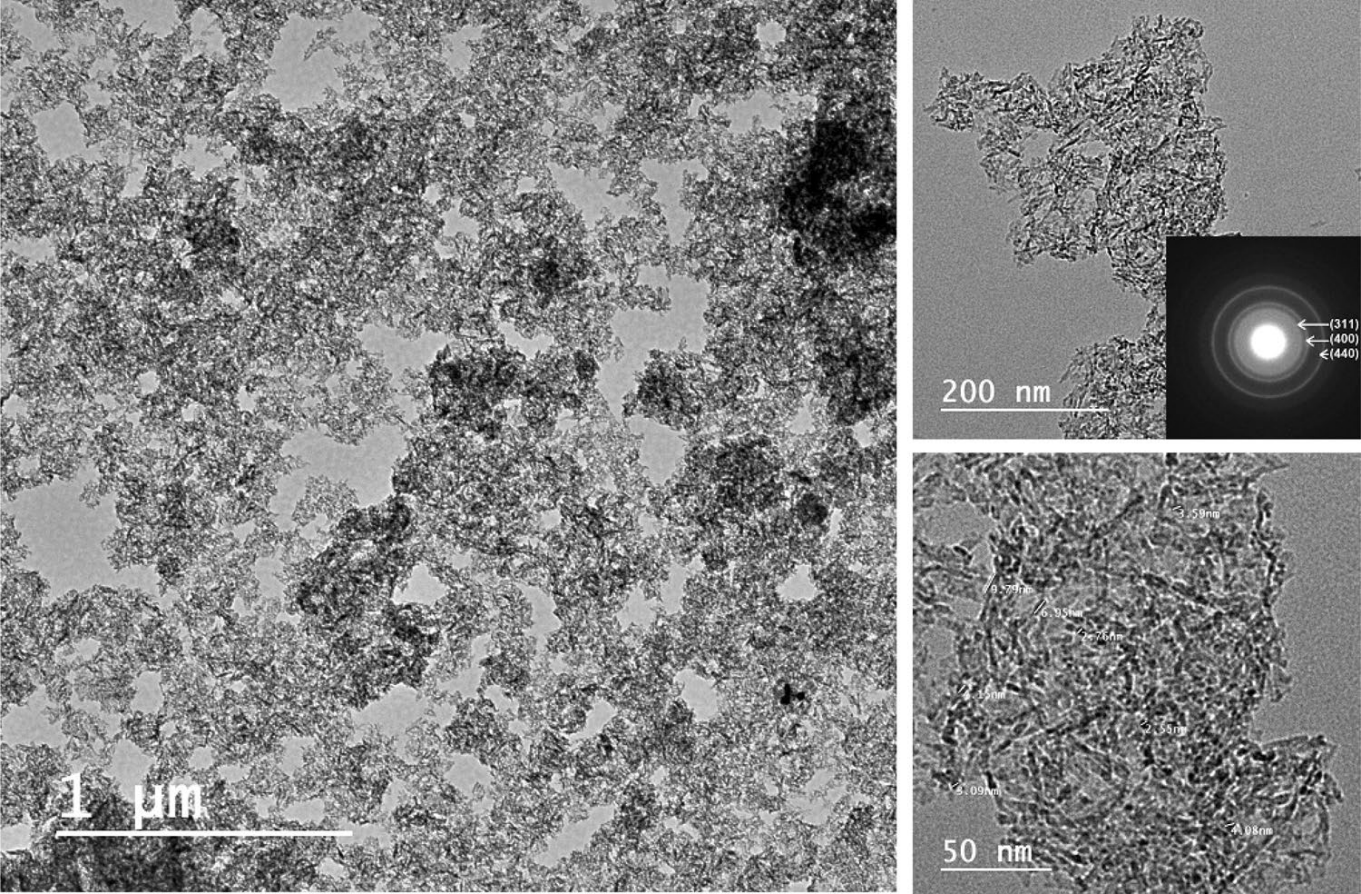
TEM micrographs of fabricated mesoporous γ-Al_2_O_3_NPs at various magnifications.

The morphology and particle size of the mesoporous γ-Al_2_O_3_NPs were analyzed via TEM microscopy as shown in (Fig. 4). A scaffold-like or lathlike configuration, comparable to the mesoporous alumina made from aluminum isopropoxide, with non-ionic block copolymer P123 serving as the structure-directing template in an acidic aqueous environment, can be seen^52^. Similarly, Samain et al. produced two highly porous γ-Al_2_O_3_NPs by calcining boehmite and amorphous aluminum (oxy)hydroxide using a sol–gel-based technique, and they obtained equivalent TEM micrographs^53^. No discernible ordering of the pore layout can be found. Particles in the size range of 2.55–9.79 nm can be observed forming aggregates with gaps or pores. The worm channels or sponge-shaped pores that might result from entangled nano-lattices indicate a highly porous system with a strongly interconnected porous structure. Commonly, to get the high pore volumes and surface areas, the mesopores are believed to have good connectivity, particularly when combined with the nitrogen sorption studies discussed above. Additionally, from the diffusion point of view, this type of interconnected pore is supposed to be beneficial for adsorption and catalysis applications. Thus, according to their morphology and small sizes, the implementation of mesoporous γ-Al_2_O_3_NPs can be expected to be prominent in TFN membranes. The inset selected area electron diffraction (SAED) in Fig. 4 also displays three concentric rings corresponding to the diffraction planes of (311), (400), and (440). This is also consistent with the data obtained from the XRD analysis of the γ-Al_2_O_3_ phase.

**Figure 5:**
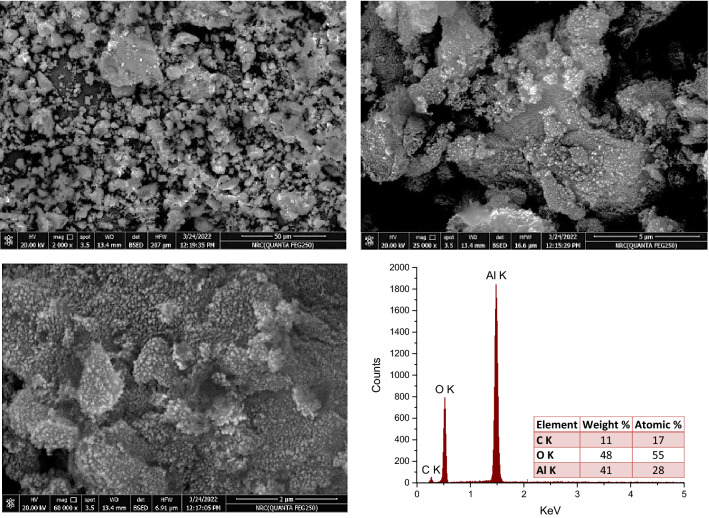
SEM micrographs at various magnifications and EDX analysis of fabricated mesoporous γ-Al_2_O_3_NPs.

Figure 5 depicts surface morphological features of fabricated mesoporous γ-Al_2_O_3_NPs at various magnifications. Some sponges and heterogeneous assemblies can be observed within the SEM images at a low magnification, which can be explained on the basis of possible phase shifts that occur during the sequential calcination and drying processes^54^. Figure 5, SEM image at higher magnification, shows the spherical shapes of the NPs. These results are consistent with the TEM, DLS, and Nitrogen adsorption–desorption isotherms analysis averages of 2.55–9.79 nm, 28.9 nm, and 15.87 nm, respectively. Moreover, EDX element analysis showed that the fabricated mesoporous nanoparticles consist mainly of aluminum and oxygen atoms with some carbon atoms. The disclosure of carbon atoms is consistent with the earlier findings in the FTIR spectrum of the potential covalently bonded carboxylate groups (comprise bridging carboxylate) covering the γ-Al_2_O_3_NPs surface.

### Characterization of TFN-Al_2_O_3_ membranes

**Figure 6:**
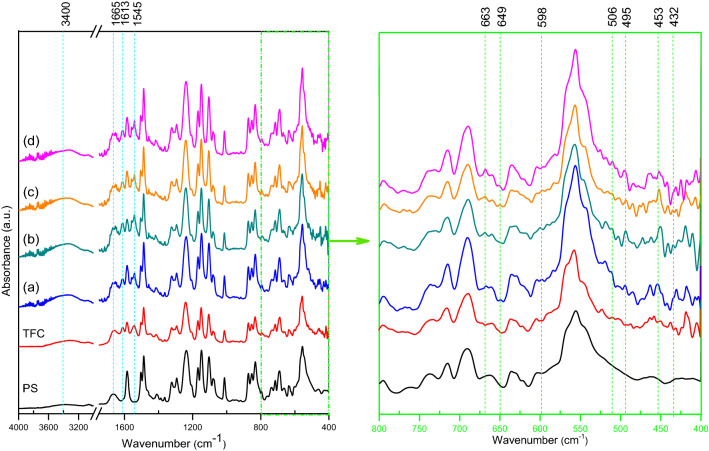
ATR-FTIR of PS, TFC and (**a**–**d**) TFN-Al_2_O_3_ membranes with varied fractions of mesoporous γ-Al_2_O_3_NPs (0.01, 0.03, 0.06, and 0.15 wt.%, respectively).

The efficiency of the interfacial polymerization of the PA/Al_2_O_3_NPs composite was demonstrated by ATR-FTIR analysis. The presence of a broad absorption peak in all spectra under study at around 3400 cm^−1^ is attributed to the stretching vibrations of the –OH groups from water molecules^55,56^. When comparing the spectra of the PS, TFC, and TFN-Al_2_O_3_ membranes, similar PA bands with slight shifts are clearly observed in the TFC and TFN-Al_2_O_3_ membranes (Fig. 6). The strong bands observed at 1665 cm^−1^, 1613 cm^−1^, and 1545 cm^−1^ could be assigned to the C = O groups of amides, aromatic PA rings, and the vibrational bending mode of N–H groups in amides, respectively^12,57^. This confirms the success of the interfacial polymerization process in the fabricated TFN sheets^58^. In TFN-Al_2_O_3_ membranes, on the other hand, new peaks in the fingerprint region appear in the range of 1000–400 cm-1, and their intensity increases with increasing content of mesoporous γ-Al_2_O_3_NPs, which are assigned to the stretching frequencies of the O–Al-O and Al–OH groups. This in turn confirms the presence of mesoporous γ-Al_2_O_3_NPs embodied in the active thin layer of PA^59,60^. Further, the absorption band at 649 cm^−1^, the low band in the FTIR spectrum of the TFN-Al_2_O_3_ membrane, is attributed to the bending vibrations of the Al–O–Al bonds. Over and above, the octahedral coordination of the Al^3+^ ions can still be confirmed by the new peak that appeared at 663 cm^−1^ ^40^.

**Figure 7:**
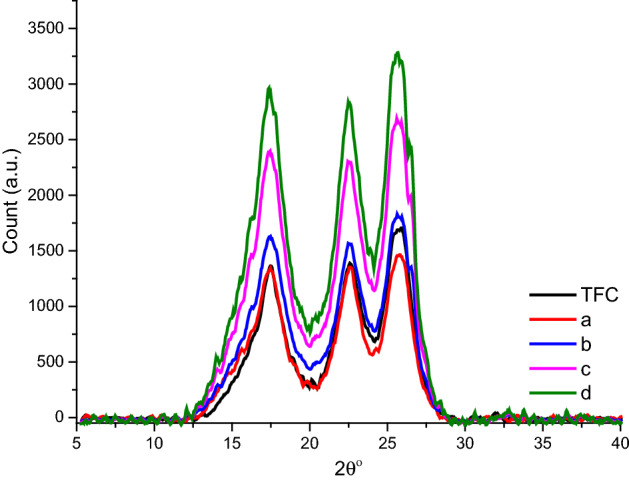
XRD profiles of TFC and (**a**–**d**) TFN- Al_2_O_3_ membranes with varied fractions of mesoporous γ-Al_2_O_3_NPs (0.01, 0.03, 0.06, and 0.15 wt.%, respectively).

XRD analysis was performed to further investigate the effect of mesoporous γ-Al_2_O_3_NPs embedded in the PA layer on the crystalline shape of the fabricated membranes. The XRD profiles of TFC and TFN-Al_2_O_3_ membranes with different fractions of mesoporous γ-Al_2_O_3_NPs (0.01, 0.03, 0.06, and 0.15 wt.%) are shown in Fig. 7 and Table 2. The peaks in the XRD profile of the TFC membrane are attributed to the semicrystalline polyester fabric support^61^. As can be noted, the diffraction peaks from the γ-phase of Al_2_O_3_NPs cannot be seen. Additional diffraction peaks appeared in TFN-Al_2_O_3_ membrane spectra (Fig. 7b,c,d), with intensities increasing with increasing contents of mesoporous γ-Al_2_O_3_NPs, which is likely due to the incorporation of mesoporous γ-Al_2_O_3_NPs in the PA layer. Similarly, the shift of the diffraction peaks to lower 2-theta angles occurred by increasing the content of mesoporous γ-Al_2_O_3_NPs from 0.01 to 0.15%.

**Figure 8:**
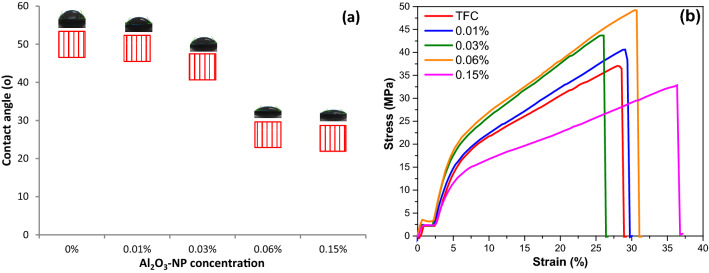
(**a**) Water contact angle and (**b**) stress–strain curves of TFC membrane, and TFN-Al_2_O_3_ membranes with varied fractions of mesoporous γ-Al_2_O_3_NPs.

Further, to explore the effect of mesoporous γ-Al_2_O_3_NPs on the surface hydrophilicity of TFC films, the contact angle of the water droplet was measured on the TFC membranes loaded with different concentrations of mesoporous γ-Al_2_O_3_NPs (Fig. 8a and Table 1). As can be seen, the addition of hydrophilic Al_2_O_3_NPs and increasing its concentration in the TFC films reduces the contact angle from 50.00° to 25.3°. This decrease in the contact angle is consistent with the intrinsic hydrophilicity of Al_2_O_3_NPs, as the (OH) groups of Al_2_O_3_NPs adsorb the water molecules, which leads to an increase in the hydrophilicity of the TFN membranes. So, since the increased hydrophilicity of the membrane allows more water to pass through the membrane pores during the nanofiltration approach, i.e., the hydrophobicity of the TFC membrane decreases, which leads to an improvement in the flow of water across the TFN membranes^62^.

**Table 1:** Parameters of contact angle, mechanical, porosity, roughness, XRD, and Zeta potential of different fabricated TFN membranes.

	Mesoporous γ-Al_2_O_3_NPs concentration (wt.%)				
	0	0.01	0.03	0.06	0.15
Average contact angle (°)	50.00	48.95	44.08	26.28	25.3
Stress (MPa)	39.21 ± 1.99	41.19 ± 6.37	41.77 ± 1.19	47.58 ± 2.34	33.13 ± 2.09
Strain (%)	29.90 ± 1.83	26.51 ± 2.42	23.51 ± 4.23	32.44 ± 6.34	34.20 ± 4.01
Young's modulus (MPa)	572.43 ± 16.9	628.02 ± 18.5	658.94 ± 10.8	740.21 ± 26.7	546.36 ± 57.6
Mean pore size )nm)	27	29	31	32	23
RMS roughness, Sq (nm)	87.67	54.77	55.86	44.69	37.69
Mean roughness, S_a_ (nm)	69.32	43.84	44.48	35.01	28.60
Porosity (μm^3^)	3370.86	5996.69	6038.79	6998.79	4294.60
Zeta potential (mV)	− 15.63	− 5.59	− 10.55	− 14.19	− 19.34
2-Theta (°)	7.485	4.388	4.168	14.141	14.121
	–	–	16.269	16.242	16.222
	–	17.368	17.467	17.44	17.42
	22.657	22.599	22.584	22.557	22.537
	25.703	25.657	25.589	25.562	25.542
	–	–	26.595	26.568	26.548

Trans-membrane compressive strength is one of the most important properties of membranes, greatly influenced by their mechanical strength. The results in Fig. 8b and Table 1 show that TFC membrane samples loaded with mesoporous γ-Al_2_O_3_NPs up to 0.06 wt.% have higher tensile strength and Young's modulus than those of pristine TFC, indicating their homogeneous dispersion in the reaction system during preparation. So, when an external force is applied to the polysulfone paper, more positive stress is transferred to the PA composite matrix incorporated by mesoporous γ-Al_2_O_3_NPs without dissipation. These improvements in mechanical strength can be well correlated with the reduced porosity of TFC films incorporated with mesoporous γ-Al_2_O_3_NPs (Table 1)^63^. Similar results for the improvement of Young's modulus in previous works occurred after the addition of hydrophilic materials due to the modification of the membrane structure^64,65^. However, when the mesoporous γ-Al_2_O_3_NPs concentration is increased to 0.15 wt.%, the tensile strength and Young's modulus decrease due to the potential agglomeration that occurs at a high loading of the mesoporous γ-Al_2_O_3_NPs. This results in brittle spots in the membrane and causes large cavities to appear in the surface and/or pores in the sub-layers of the fabricated membranes^65^. Conversely, the incorporation of mesoporous γ-Al_2_O_3_NPs into the TF layer first reduces the elongation percentage up to 0.03 wt.% and then increases it again with increasing mesoporous γ-Al_2_O_3_NPs concentration. Further, interfacial interactions between the PA polymer and mesoporous γ-Al_2_O_3_NPs via electrostatic contact and hydrogen bonding are also responsible for the TFC mechanical strength characteristics^63^. Thus, from the mechanical test results, TFC membranes combined with Al_2_O_3_NPs can resist high forces and pressures during the filtration process. The data obtained in this study is similar to what was obtained earlier^66^.

Phase analysis light scattering (PALS) was used to determine the zeta potentials of the TFC and TFN membrane surfaces at pH = 7. Table 2 demonstrates that the negatively charged zeta potentials of all the manufactured TFC and TFN membranes vary. Several variables affect the negative zeta potential charges. One of these is related to the Al^+3^ in Al_2_O_3_NPs' potent capacity to withdraw electrons from the copious carboxyl groups in the polyamide matrix, which would ultimately cause a drop in the negative charge density during the interfacial polymerization process^67^. The unreacted acyl groups of trimesoyl chloride's carboxyl groups, which will increase the membrane's surface negative charge, are the other significant element. However, there may be a decrease in the density of the negative charge on the membrane's surface due to a probable interaction between those acyl groups and the hydroxyl groups of Al_2_O_3_NPs^68^. Some of the embedded Al_2_O_3_NPs may also reside on the membrane's surface, where Al_2_O_3_NPs in close proximity to the electrolyte may cause the membrane's surface to take on an additional negative charge^69^. Similar outcomes were reported in a prior study using interfacial polymerization to embed cellulose nanocrystals in polyamide layers^54^.

**Table 2:** Treatment efficiency of the different membranes for real brackish water.

Parameter	Influent (mg/L)	Effluent from TFC		Effluent from TFN-Al_2_O_3_	
		(mg/L)	R %	(mg/L)	R %
pH	8.0	8.2	–	8.1	–
TDS	5455	1576	71.10	1016	81.37
Ca^2+^	43	16.0	62.79	16.0	62.79
Mg^2+^	143	31.0	78.32	20.3	85.80
Na^+^	1702	502.0	70.50	190	88.83
K^+^	52	29.0	44.23	28	46.15
CO_3_^2−^	N.D*	N.D*	N.D*	N.D*	N.D*
HCO_3_^−^	51	28	45.09	28	45.09
SO_4_^2−^	1490	402	73.02	160	89.26
Cl^−^	2002	602	69.93	280	86.01
NaCl	3400.95	1020.39	69.99	850.00	75.00
Na_2_SO_4_	1130.55	129.00	88.58	40.00	96.46
MgSO_4_	710.68	152.09	78.59	40.66	94.27
Mg(HCO_3_)_2_	72.03	15.00	79.17	12.00	83.34
Ca(HCO_3_)_2_	71.02	45.06	36.55	14.03	80.24

*N.D Not detected.

**Figure 9:**
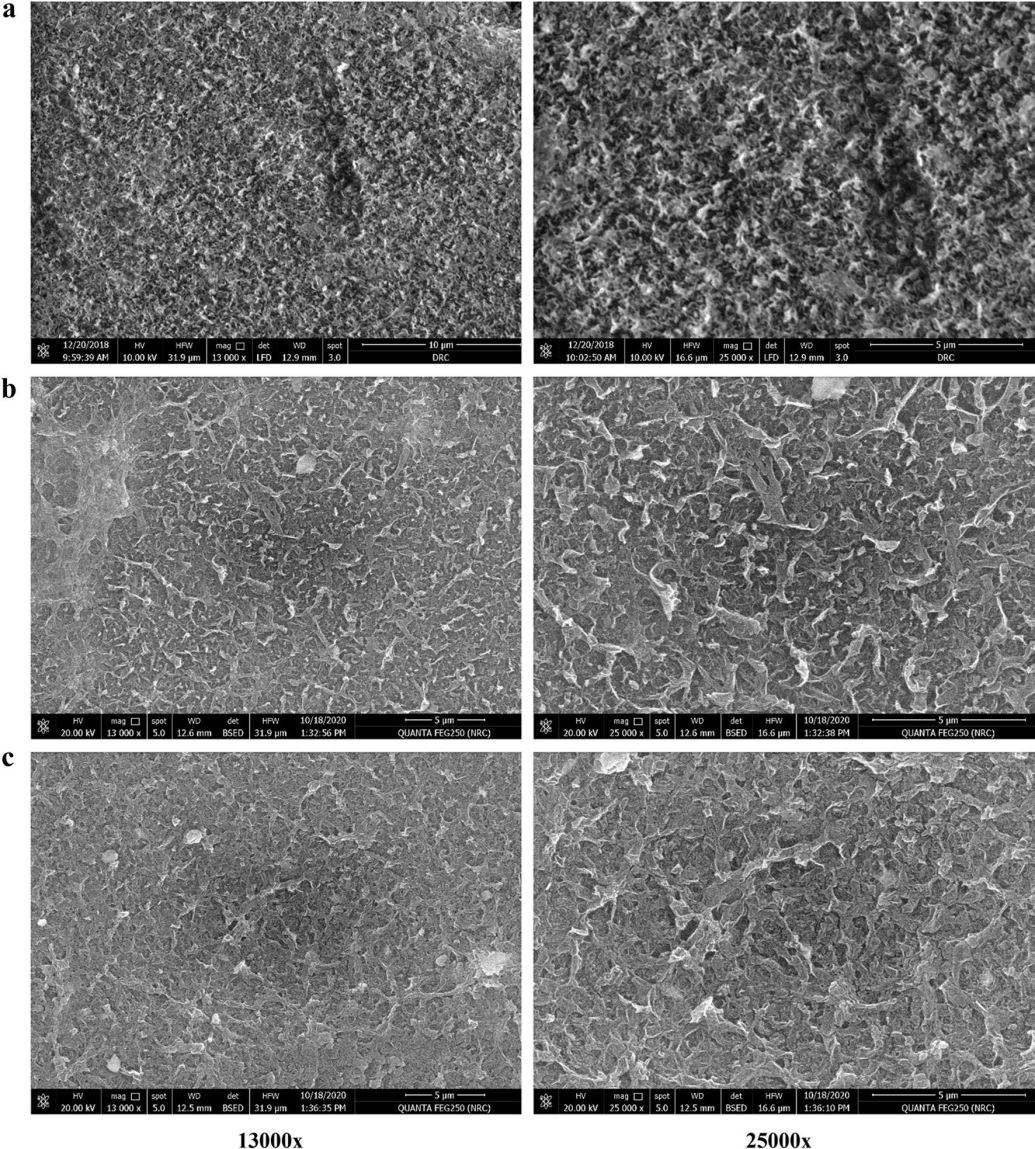

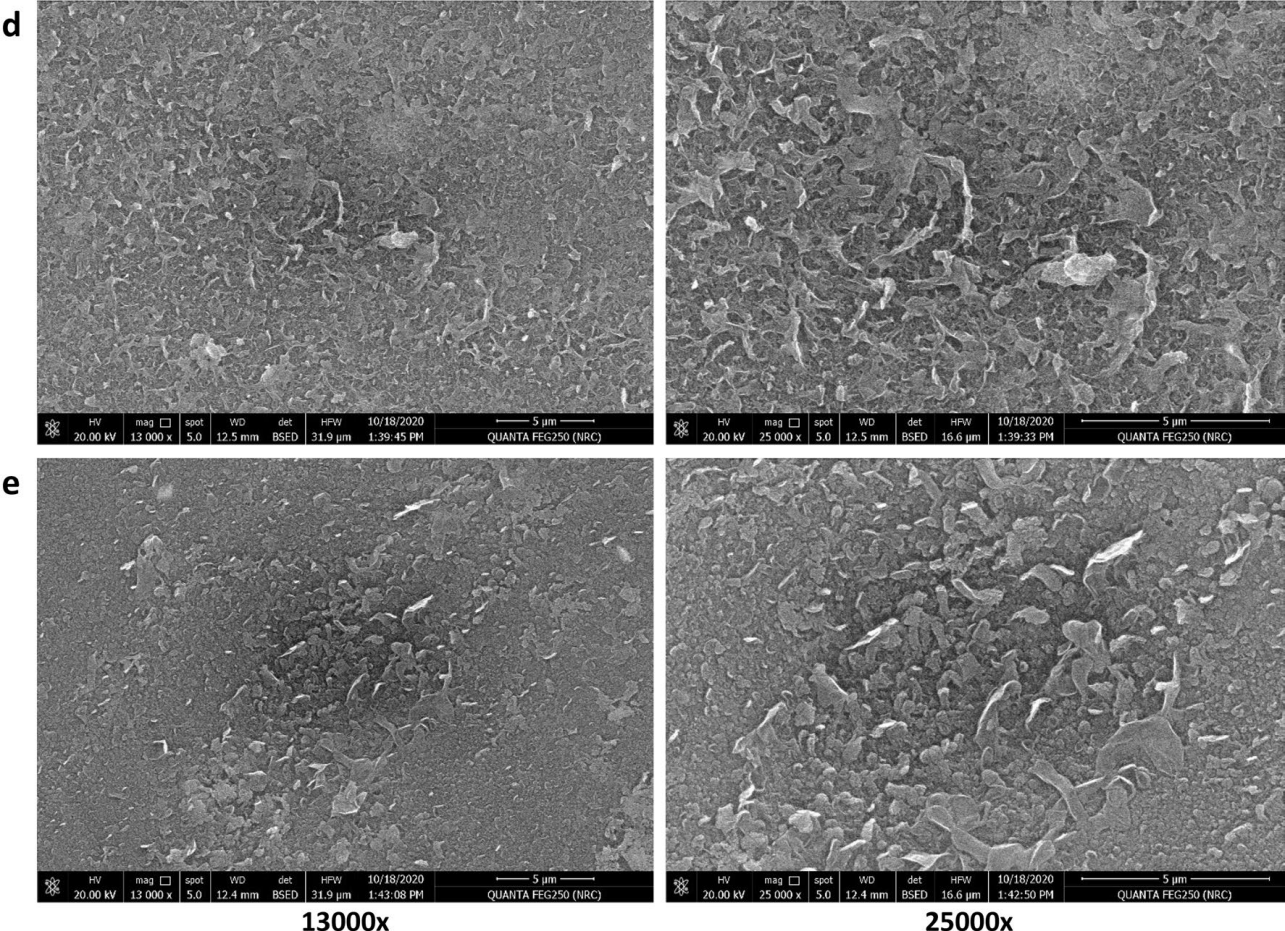
SEM micrographs of (**a**) TFC membrane, and (**b**–**e**) TFN-Al_2_O_3_ membranes with varied fractions of mesoporous γ-Al_2_O_3_NPs (0.01, 0.03, 0.06, and 0.15 wt.%, respectively).

**Figure 10:**
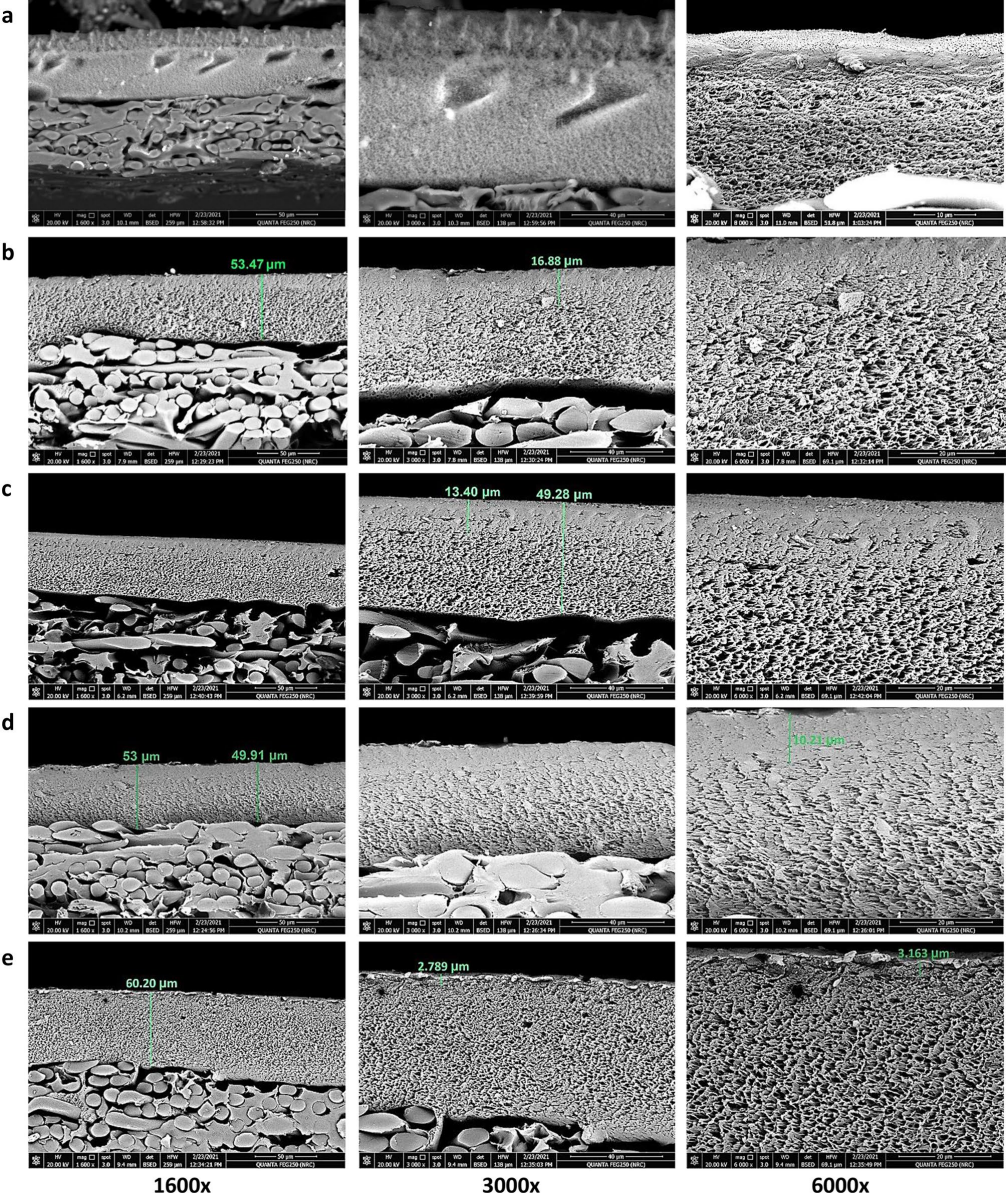
SEM cross-section micrographs of (**a**) TFC membrane, and (**b**–**e**) TFN-Al_2_O_3_ membranes with varied fractions of mesoporous γ-Al_2_O_3_NPs (0.01, 0.03, 0.06, and 0.15 wt.%, respectively).

Figures 9 and 10 show SEM micrographs of the surface and cross-section of TFC, TFN-Al_2_O_3_ membranes with varying fractions of mesoporous γ-Al_2_O_3_NPs (0.01, 0.03, 0.06, and 0.15 wt.%). Surface morphological qualitative explorations at two different magnifications of the fabricated TFN membranes ascertained that intercalation of mesoporous γ-Al_2_O_3_NPs into TFN films can enhance their surface smoothness and change the up-and-down structure of the TFC membrane. SEM micrographs also show that the prepared TFN-Al_2_O_3_ membranes have a smoother surface and that the detachment of mesoporous γ-Al_2_O_3_NPs added at the interface was not observed. This homogeneous distribution of mesoporous γ-Al_2_O_3_NPs is likely related to the carbon-building modes of polysulfone and PA that facilitate the fine dispersion of mesoporous γ-Al_2_O_3_NPs in the membrane mold without any aggregation of the nanocomposites^70^. In addition, the cohesive membrane structure overwhelms any brittleness and controls any negative effects on membrane stability. Although the concentrations of mesoporous γ-Al_2_O_3_NPs loaded in the TFN matrix increase to 0.15 wt.% and some agglomerates appear, the surface remains much smoother when compared to the pristine TFC membrane. These results are in agreement with those discussed in the mechanical strength section.

From SEM micrographs of the fabricated TFC membrane cross-section in Fig. 10, we can observe an upper dense layer with a finger-print structure consistent with polysulfone polymer in the TFC membrane. Conversely, the cross-sectional view of TFN-Al_2_O_3_ membranes revealed a uniformly distributed asymmetric structure possessing a very fine and dense layer with almost imperceptible pores on top of the porous supporting layer, which roughly covers the cross-section of the films. Similar forms of the porous structure as in Fig. 10b–e have been observed in other related prior studies^71,72^. The polysulfone substrates' sponge-like skin layers give the polyamide rejection layers a smooth surface and enough mechanical cushions. As can be seen in the SEM images (Fig. 10), for TFN-Al_2_O_3_ membranes, the presence of hydrophilic mesoporous γ-Al_2_O_3_NPs within the interfacial polyamide layer decreases the higher-density sponge polysulfone layer to 3 μm, leading to the formation of a large number of interlinked tiny pores that increase the mass transfer area for the TFN-Al_2_O_3_ membrane. Consequently, boosting the membrane's wettability and porosity improves flux rates and performance constancy^73,74^. In light of this, compared to the pristine TFC membrane, the pores of the mesoporous γ-Al_2_O_3_NPs-incorporated membrane appeared to be smaller. Thus, the mesoporous γ-Al_2_O_3_NPs addition reduces the size of the pores that resembled sponges. The small pores are created by the solid–liquid phase separation, whereas the large pores are created by the liquid–liquid phase separation^75^.

The occurrence of this framework could be the result of a self-synthesizing reaction between the amine, carbonyl, and carboxylic groups in the matrix of PA and mesoporous γ-Al_2_O_3_NPs. Several proposed pathways could occur simultaneously during this reaction mechanism, as illustrated in Fig. 1b. One of them is the single assignment of Al^3+^ ions to the carbonyl groups by attaching nitrogen atoms to the oxygen atoms of Al_2_O_3_NPs and the double assignment of Al^3+^ ions towards the carboxyl group in the PA layer. Also, the interfacial hydroxyl groups attached to the mesoporous γ-Al_2_O_3_NPs can form hydrogen bonds with the carboxyl groups^23^. Thus, the incorporation of mesoporous γ-Al_2_O_3_NPs within the TFN layer greatly affects the construction of the membranes. As the incorporated mesoporous γ-Al_2_O_3_NPs accelerate the mass transfer functions between the non-solvent and the organic solvent in the polymerization approach, resulting in the creation of larger pore sizes for outstanding rapid mass transfer performance^23^. Furthermore, the symmetric distribution of Al_2_O_3_NPs in the TFN membrane can be inferred from the absence of mesoporous γ-Al_2_O_3_NPs clumping in the cross-section images. As previously reported, rapid diffusion of mesoporous γ-Al_2_O_3_NPs in trimesoyl chloride solution leads to interaction with trimesoyl chloride and M-phenylenediamine monomer in the polysulfone backing sheet during the interfacial polymerization process, resulting in a wider pore size than the pristine TFC membrane^76^. Moreover, the morphology and surface roughness of the polysulfone support layer can lead to the heterogeneous filling of the M-phenylenediamine monomer on the membrane interface, which can create a PA layer with inactive shapes that is thick and relatively rough^77^. Fortunately, in this study, the incorporation of mesoporous γ-Al_2_O_3_NPs into the TF membrane formed TFN membranes with hydrophilic properties, larger pore sizes, and smoother surfaces (Table 1).

In addition, the decrease in the number of acyl chloride groups in the Trimesoyl chloride monomer, due to its interaction with the O–H groups attached to Al_2_O_3_NPs, reduces the thickness of the PA layer in the TFN-Al_2_O_3_ membranes. This leads to the formation of an active, pure, homogeneous, and thin layer of PA^78^. Compared with other TFN-Al_2_O_3_ concentrations, although 0.03 wt.% has a medium layer thickness of PA, moderate hydrophilicity, and higher roughness, these values are still lower than those of the TFC membrane and may be compensated by the larger surface pore mean size and porosity. Similar results have been reported in previous studies^11^. Moreover, the increase in water permeability while decreasing the thickness of the nano-layer is attributed to the shrinkage of the water transport pathways^79^.

**Figure 11:**
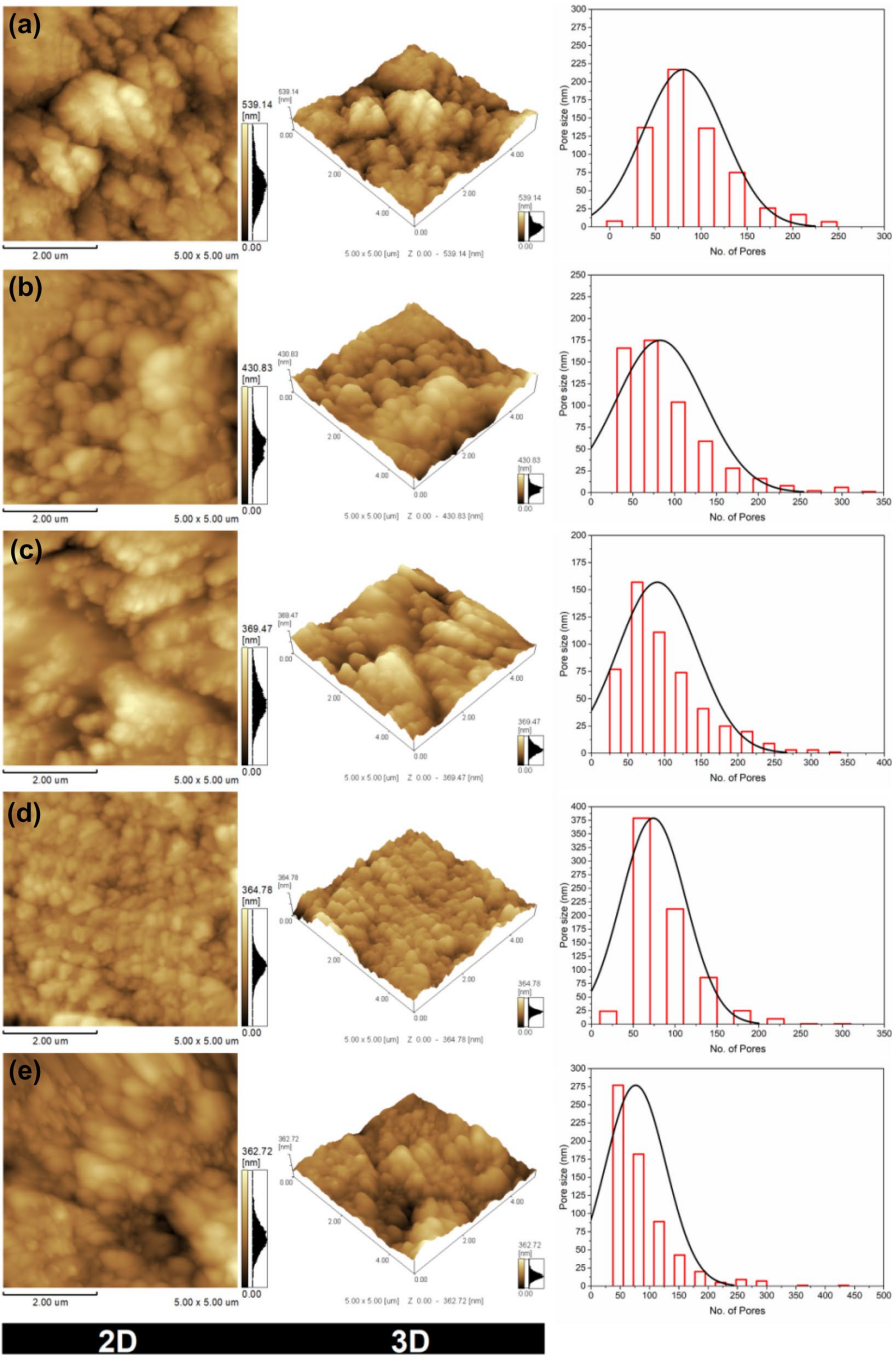
AFM 2 and 3 dimensional topography and pore size distribution of (**a**) TFC membrane, and (**b**–**e**) TFN-Al_2_O_3_ membranes with varied fractions of mesoporous γ-Al_2_O_3_NPs (0.01, 0.03, 0.06, and 0.15 wt.%, respectively).

In addition to the morphological studies, a two- and three-dimensional topographical AFM survey was performed to determine the porosity, root mean square roughness (S_q_, nm), surface roughness (S_a_, nm), mean pore size, and pore size distribution as shown in Fig. 11 and Table 1. The two values of S_a_ and S_q_ of the TFC membrane are 69.32 nm and 87.67 nm, correspondingly. The S_a_ and S_q_ values of TFN-Al_2_O_3_ membranes are lower than those of the pristine TFC membrane due to the mesoporous γ-Al_2_O_3_NPs embedded. Accordingly, the residual M-phenylenediamine monomer that remains trapped in the polysulfone support pores during interfacial polymerization processes could be the cause of the low pore size of the pristine TFC membrane (Table 2). In addition, the thicker and coarser layout of the created TFC membrane may lead to ineffective PA film formation^77^. On the contrary, TFN membranes synthesized with varying amounts of mesoporous γ-Al_2_O_3_NPs have more hydrophilic properties, a smoother surface, and a larger pore diameter. Therefore, an even PA layer was forged without any sedimentation or weakening defects^78^.

Likewise, the findings show that the pristine TFC membranes' surface morphologies underwent significant alteration after the addition of mesoporous γ-Al_2_O_3_NPs. S_q_ and S_a_ significantly decreased, and the surface of the mesoporous γ-Al_2_O_3_NPs embedded membrane seemed to be smoother as a result. The tiny area, large quantity patterns took the place of the steep ups and downs with large areas and small numbers. The morphology of the membrane surface changed due to the inclusion of mesoporous γ-Al_2_O_3_NPs. This affected the shape, size, and numbers of pores on the membrane surface, resulting in more uniform pores with smaller sizes, as were shown by the SEM pictures. The well-dispersed mesoporous γ-Al_2_O_3_NPs inside the substrate is likely what caused the decrease in the substrate surface roughness after its embedding^80^. Similar findings of roughness decline were previously reported when embedding varied concentrations of graphene oxide nanoplates in polysulfone membranes^81^.

As a result, the PA coating in the TFN-Al_2_O_3_ nanofiltration membrane become smoother as the mesoporous γ-Al_2_O_3_NPs content increases. Even the specific surface topographic feature (Z-band) was 539.14, 430.83, 369.47, 364.78, and 362.72 nm for mesoporous γ-Al_2_O_3_NPs contents of 0, 0.01, 0.03, 0.06, and 0.15 wt.%, respectively. The possible explanation for such a Z-band difference is the fine dispersion of Al_2_O_3_NPs through the PA layer and the type of bonding created. On the other hand, the pore size increases until it reaches its maximum value at 0.06 wt.% mesoporous γ-Al_2_O_3_NPs and then decreases again with the addition of more mesoporous γ-Al_2_O_3_NPs content. Hence, a 0.03 wt.% concentration of mesoporous γ-Al_2_O_3_NPs can be proposed, which has the larger surface pore diameter with moderate hydrophilicity and surface roughness, as the optimum concentration of mesoporous γ-Al_2_O_3_NPs. These results are consistent with what has been described in preceding studies^82^.

**Figure 12:**
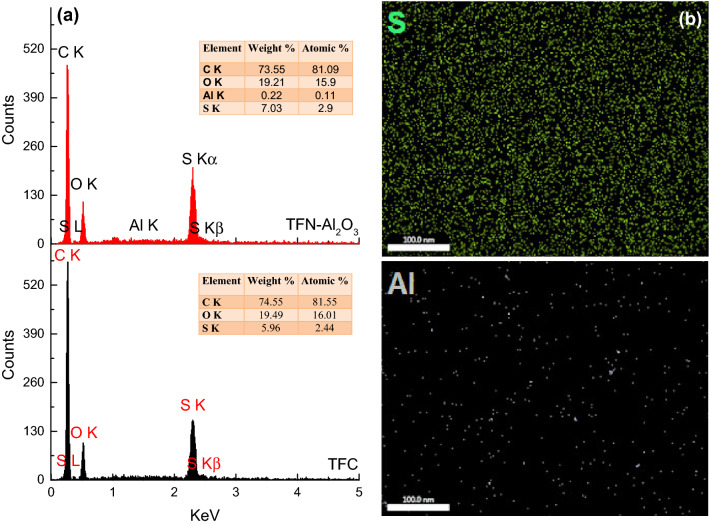
EDX analysis of TFC and 0.03 TFN-Al_2_O_3_ membranes (**a**) and EDX mapping of 0.03 TFN-Al_2_O_3_ membranes showing the distribution of sulfur and aluminum.

Finally, the EDX analysis of TFC and TFN-Al_2_O_3_ membranes (with the mesoporous γ-Al_2_O_3_NPs optimum concentration of 0.03 wt.%) reveals the carbon, oxygen, sulfur, and aluminum (if any) contents (Fig. 12a). The existence of the aluminum element supported the successful integration of mesoporous γ-Al_2_O_3_NPs onto the TFN membrane surface. Figure 12b shows the EDX mapping of sulfur and aluminum elements in a 0.03 wt.% TFN-Al_2_O_3_ membrane where a relatively homogeneous distribution of aluminum across the membrane surface can be noticed. Thus, the even dispersion of mesoporous γ-Al_2_O_3_NPs in the organic phase and high density within the internal structure of the polyamide matrix would lead to an enhancement of both water flux and rejection performance^83^.

### Separation performance of the pristine TFC and TFN-Al_2_O_3_ membranes

#### *Separation performance at different concentrations of mesoporous γ-Al*_*2*_*O*_*3*_*NPs*

**Figure 13:**
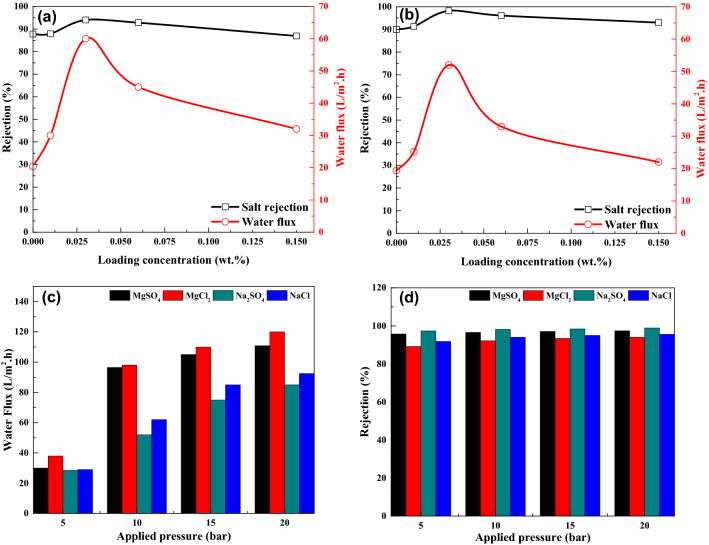
Permeate flux and rejection results of (**a**) NaCl and (**b**) Na_2_SO_4_ at different loading concentration of mesoporous γ-Al_2_O_3_NPs. Variations of water flux (**c**) and salt rejection (**d**) of 0.3 wt.% TFN-Al_2_O_3_ membrane with operating pressure (2 g/L inorganic salt aqueous solutions).

The salt rejection of the TFN-Al_2_O_3_ membrane was studied using ionic solutions of monovalent NaCl and divalent Na_2_SO_4_. The results represented in Fig. 13a,b show that increasing the concentration of mesoporous γ-Al_2_O_3_NPs up to 0.03 wt.% in the casting solution improves the rejection of NaCl and Na_2_SO_4_ for the fabricated nanofiltration films. This may be attributed to the adsorption property of the mesoporous γ-Al_2_O_3_NPs, which resulted in superior interactions between the membrane matrix and the ions^84^. Subsequently, the rejection decreases by increasing the concentration of mesoporous γ-Al_2_O_3_NPs from 0.03 to 0.15 wt.% in the membrane matrix. This can be attributed to the agglomeration of NPs at a high additive concentration (see Fig. 11, AFM image, e), which reduces the amount of adsorptive active sites/active surface area, and thus diminishes the salt adsorption capacity by the synthesized nanocomposite and increases percolation of ions throughout the membrane matrix^85^. The results in Fig. 13 show that increasing the concentration of mesoporous γ-Al_2_O_3_NPs in the organic phase solution to 0.03 wt.% increases the water flux of the as-fabricated TFN-Al_2_O_3_ membrane to 60 and 50 L/(m^2^ h) for NaCl and Na_2_SO_4_ salts, respectively. Further, two main factors affecting the membrane permeation flux are membrane hydrophilicity and morphology^86^. It is well known that rising membrane hydrophilicity improves the permeation flux^87^. In addition, membrane porosity, increased pore size, and reduced surface thickness are morphological parameters that can improve membrane permeable flux as well. Thus, the improved water flux in Fig. 13 could be partly due to the increased membrane aqueous affinity/hydrophilicity that enhances water flux. Another rationale for improved water flux is the larger pores and voids in the membrane structure and more porosity caused by the addition of mesoporous γ-Al_2_O_3_NPs in the organic phase solution (0.01 and 0.03 wt.%) (as observed in Figs. 9 and 10), which facilitates water transfer across the membrane. Conversely, when 0.06 and 0.15 wt% mesoporous γ-Al_2_O_3_NPs are added to the TFN membrane, water flux for NaCl and Na_2_SO_4_ salts is reduced to [45 and 32 L/(m^2^.h)] and [33 and 22 L/(m^2^.h)], respectively. This decrease in flux may be due to several factors that could be related to the phenomenon of pore filling/clogging at high concentrations of NPs, reducing the movement of water that others have discovered^88,89^. Besides various features such as surface pore size, hydrophilicity, and surface morphology that control the flux mechanism as we discussed earlier, these factors can influence the pore geometry of the modified membrane and thus control membrane flux^90^. To our knowledge, no study in the literature has been found for the use of mesoporous γ-Al_2_O_3_NPs incorporated with TFN membrane for desalination purposes; however, Kotp has used Al_2_O_3_NPs, biosynthesized via camphor extract, integrated with TFN membrane for desalination purposes and reported a water flux of 69 L/(m^2^.h)^33^.

#### *Separation performance of TFN-Al*_*2*_*O*_*3*_* membranes at different operation pressure*

As shown in Fig. 13, the permeate flux of TFN-Al_2_O_3_ membranes was calculated and plotted against the applied trans-membrane pressure (c and d). The flow of the TFN-Al_2_O_3_ membrane increases with increasing the applied pressure, up to 20 bar. The findings of Zhu et al. show that even with an increase in the applied pressure, the greater pressure boosts the permeability of water across the membrane to a steady state without any other changes, supporting the results shown in Fig. 13c,d ^91^. In the current study, the representation of dissolution and diffusion successfully depicted water permeation across the 0.3 wt.% TFN-Al_2_O_3_ membrane of various inorganic salts versus applied pressure as reported by Sun et al. (Eq. 5):

5 $$F_{W} = A\left( {\Delta P - \Delta \pi } \right)$$

Here, penetration flux *(F*_*W*_*)*, operating pressure difference *(∆P)*, and flux penetration coefficient *(A)*^92^.

As shown in Eq. (5), the solute rejection is related to two parameters: the convection coefficient and the diffusion coefficient. The convection coefficient is only correlated to the osmotic pressure, while the diffusion coefficient is correlated to the pressure and concentration of the solution. Under low working pressure, the diffusion coefficient and convection coefficient together function on the membrane in the filtration operation. At high pressure, the convection coefficient has the maximum effect. Therefore, an increase in the operating pressure will definitely cause an increase in the flux of diverse salt solutions. When 2 g/L of different inorganic salt solutions of NaCl, Na_2_SO_4_, MgCl_2_, and MgSO_4_ were used, the *F*_*W*_ increased with the increase of ∆P. After applying 5 bar, all fluxes for different inorganic salts ranged from 28.5 to 38 L/(m^2^.h). The water flux increased to nearly 100 L/(m^2^.h) for MgSO_4_ and MgCl_2_ salts, while it was 52 L/(m^2^.h) and 62 L/(m^2^.h) for Na_2_SO_4_ and NaCl salt solutions, respectively. After applying 15 and 20 bar to the used membrane, there was no significant difference in the water flux for MgSO_4_ and MgCl_2,_ while the water flux for Na_2_SO_4_ was improved by 63.5% after applying 20 bar. However, it was only enhanced by 49% in the case of NaCl salt solution. Concerning the percentage of rejection, the results when using different pressures are somewhat similar, which leads to the conclusion that the use of 10 bar is the optimal condition for the use of TFN-Al_2_O_3_ membrane.

#### *Separation performance of TFC and TFN-Al*_*2*_*O*_*3*_* membranes at constant temperature (25 *°*C) and pressure (10 bar)*

**Figure 14:**
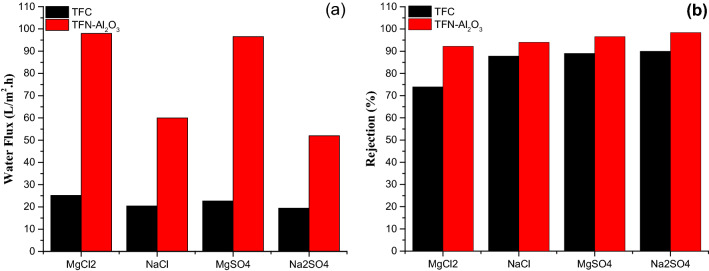
Water flux (**a**) and Salt rejection (**b**) of 0.3 wt.% TFN-Al_2_O_3_ membrane.

The water flux and different salt rejection of NF membranes doped with mesoporous γ-Al_2_O_3_NPs at 0.03 wt.% are shown in Fig. 14. As aforementioned, the shift of flux is determined by many aspects, such as the membrane hydrophilicity and thickness, bottleneck effect, the own pore of mesoporous γ-Al_2_O_3_NPs, and the interface channel between mesoporous γ-Al_2_O_3_NPs and polyamide. As shown in Fig. 14a, the flux of divalent salt (Na_2_SO_4_) solution and monovalent salt (NaCl) solution reached 45 L/(m^2^.h) and 68 L/(m^2^.h), respectively, under operating pressure (10 bar), indicating that the nanofiltration membrane is low in energy consumption and has a high practical application value. Figure 14b displays the rejection rate of the NF membrane for four types of salt solutions, following the order of Na_2_SO_4_ > MgSO_4_ > NaCl > MgCl_2_, which is consistent with the typical properties of a negatively charged NF membrane. Compared with TFC, the NF membranes modified by mesoporous γ-Al_2_O_3_NPs show a significant increase in the retention of the divalent salts Na_2_SO_4_ and MgSO_4_. This is credited to the increase in the degree of cross-linking and the density of the previously mentioned NF membranes. The main factor controlling the retention rate of NaCl is the pore size sifting effect, as monovalent salts are less influenced by the NF membrane charge. Furthermore, membrane densities have less effect on NaCl permeability than other divalent salts due to the relatively small water radius^93^. Accordingly, a thinner separation layer is advantageous for more monovalent chloride ions passing through the NF membrane with water molecules, which leads to a lower rate of NaCl penetration.

#### *Long-term performance of TFN-Al*_*2*_*O*_*3*_* membranes at constant temperature (25 *°*C) and pressure (10 bar)*

**Figure 15:**
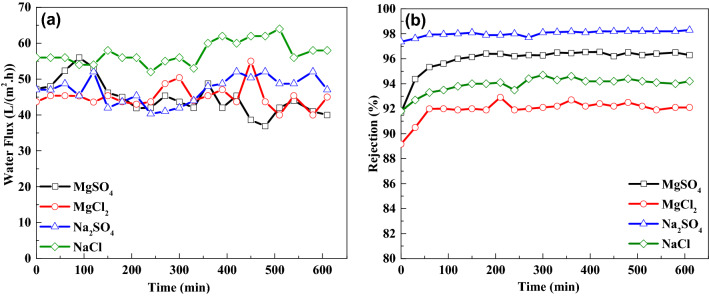
Long-term stability (**a**,**b**) of 0.3 wt.% TFN-Al_2_O_3_ membrane (with 2 g/L inorganic salt aqueous solutions at 10 bar).

Herein, we tested the stability of the 0.03 wt.% TFN-Al_2_O_3_ membrane at 10 bar when salt solutions of MgCl_2_, MgSO_4_, NaCl, and Na_2_SO_4_ are applied at concentrations of 2 g/L (Fig. 15a,b). For salt solutions of Na_2_SO_4_, MgSO_4_, NaCl, and MgCl_2_, the results show a stable flux of around 52, 96.5, 60, and 98 (L/m^2^.h), respectively, and a rejection of 98.4%, 96.5%, 92.2%, and 94%, respectively. Thus, the rejection of NaCl was increased by 5%. Moreover, even though the separation factor of NaCl/Na_2_SO_4_ decreased from 68.75 to 37.5, it is still higher than in other literature^94^. The experiment proves that a 0.03 wt.% TFN-Al_2_O_3_ membrane with high water permeability and high separation performance has prominent potential in nanofiltration separation implementations, and these results push the researchers to use this type of membrane on a pilot scale for a long time. As indicated in Table 2, the monovalent/bivalent anion selectivity of the nanofiltration membrane is enhanced, and the separation factor of the optimal 0.03 wt.% TFN-Al_2_O_3_ membrane is 68.75. To further study the potential of the NF membrane in practical applications, a real brackish water sample was collected from the Safaga area, Red Sea coast, Egypt.

Real water was utilized to feed the cross-flow system at optimum pressure. Both TFC and TFN-Al_2_O_3_ membranes succeeded in minimizing the concentration of Cl^−^ and Na^+^ ions. The Cl^−^ and Na^+^ removal efficiencies of the pristine TFC and TFN-Al_2_O_3_ membranes were (69.9%; 70.5%, and 86%; 88.8%), respectively. The permeate concentrations were 190 mg/L for sodium and 280 mg/L for chloride after using TFN-Al_2_O_3_ membranes. According to the permitted limits of the World Health Organization (i.e., 250 and 200 mg/L for Cl^−^ and Na^+^, respectively), Na^+^ reached the permitted limits while Cl^−^ was very close^95^. This confirms the high efficiency of the TFN-Al_2_O_3_ membrane compared to TFC when used for water desalination purposes. Similar results were obtained by Malaisamy et al., who stated that the negative surface charge on the membranes must be protected by cations, or else anions can easily enter the membrane pores, preserving the internal amines and allowing the cations to saturate early^96^. Furthermore, the electrostatic energy rises during liquid streaming due to the presence of more cations on the surface, causing more anions such as SO_4_^2−^ and Cl^−^ to pass through^97^. Some cations such as Na^+^ do not pass freely, and this may indicate an increased repulsive force within the membrane pores. From Table 2, the amended selectivity of both NaCl and SO_4_^n^ (S NaCl/Na_2_SO_4_ = 62.50) in the mixed salt system may be because the concentration of Na^+^ is higher than that of Cl^−^ which allows more Na^+^ ions to pass through the membrane and thus could facilitate the permeation of Cl^−^ as a result of the charge balance^98^. Accordingly, these membranes modified with mesoporous γ-Al_2_O_3_NPs are also predicted to be employed in separation implementations. Further, the TFN-Al_2_O_3_ membrane was capable of successfully removing the hardness of the applied water samples below the WHO limit compared to using merely the TFC membrane^95^.

Eventually, the performance of the TFN-Al_2_O_3_ membrane prepared in this study under optimal conditions was compared with the performance of previous membranes used for the nanofiltration process. Hence, from Table 3, we can see that the membrane prepared in this study using mesoporous γ-Al_2_O_3_NPs showed better performance compared to those prepared previously with the same chemical composition from similar materials and those with different chemical compositions prepared by other methods. From this perspective, this would make it a good candidate for desalinating brackish groundwater in Egypt.

**Table 3:** Summary of previously published nanofiltration membranes composed of Al_2_O_3_-NPs.

Nanofiltration membrane type	Method of preparing Al_2_O_3_NPs	Method of preparing composite membrane	Water flux (composite/pristine) membranes (L/m^2^.h)	Water flux boost (%)	Rejection	References
Polyamide/mesoporous γ-Al_2_O_3_NPs	Aqueous sol–gel (Surfactant-template)	Interfacial polymerization	60.00/20.40	194.17	98.2, Na_2_SO_4_	Current work
α-alumina/γ-alumina/γ-alumina-titania ceramic	Aqueous sol–gel	Dip-coating	55.00/3800	− 98.55	67.62%,Cl	^26^
γ-Al_2_O_3_ film-coated porous α-Al_2_O_3_ hollow fiber	Aqueous sol–gel	Dip-coating	20.00/na	n.a	92%, Na	^25^
Polyamide/ γ-alumina	Aqueous sol–gel (Green synthesized via camphor extract)	Interfacial polymerization	78.75/30.24	160.42	96.5, Na_2_SO_4_	^33^
PDA/PEI/Al_2_O_3_	Hydrothermal	Co-deposition	21.00/80.00	− 75	> 91%, divalent cations	^24^
CTA/Al_2_O_3_	Commercial	Phase inversion	85.20/74.40	14.52	99.8%, NaCl	^27^
TA-γ-AlOOH/PES	Aqueous sol–gel	Phase inversion	111.00/16.50	572.72	97.3%, dye	^99^
Poly(piperazineamide)/Al(OH)_3_	Commercial	Interfacial polymerization	39.0/25.24	54.52	97.1, Na_2_SO_4_	^67^
Polyamide/Al_2_O_3_	Aqueous sol–gel	Interfacial polymerization	5.00/2.80	78.57	88%, NaCl	^23^

*n.a.* not available.

## Conclusions

In the current study, mesoporous γ-Al_2_O_3_NPs were prepared by a facile method using non-ionic surfactant polysorbates-80. The effect of different concentrations of mesoporous γ-Al_2_O_3_NPs incorporated in the PA layer on the properties of the nanofiltration membrane was investigated. The results obtained from the morphological and functional parameters and the surface roughness of the prepared nanofiltration membranes showed the successful incorporation of mesoporous γ-Al_2_O_3_NPs in the TFN membrane. When compared with the TFC membrane, it was found that by increasing the content of mesoporous γ-Al_2_O_3_NPs from 0.01 to 0.15 wt.%, the hydrophilicity and water flux of the prepared nanofiltration membranes increased significantly. The nanofiltration of inorganic salts across a TFN membrane, with a content of 0.03 wt.% mesoporous γ-Al_2_O_3_NPs, was also improved under working conditions of 2 g/L solution at 25 °C and 10 bar. Besides, the TFN-Al_2_O_3_ membrane showed a remarkable ability to reject more than 90% NaCl compared to the TFC membrane. Hence, the higher flux and higher salt rejection of the TFN-Al_2_O_3_ membrane in comparison with the TFC membrane proves that the mesoporous γ-Al_2_O_3_NPs have an important role in raising both the membrane hydrophilicity and the surface negativity. In conclusion, the current work is to produce high quality water as well as promote nano-filtration applications in remote areas with a scarcity of potable water and coastal tourist areas (e.g., hotels and beaches).

## Data Availability

All data generated or analysed during this study are included in this published article.
